# Intravenous Paracetamol Reduces Postoperative Opioid Consumption after Orthopedic Surgery: A Systematic Review of Clinical Trials

**DOI:** 10.1155/2013/402510

**Published:** 2013-11-06

**Authors:** Bright Jebaraj, Souvik Maitra, Dalim Kumar Baidya, Puneet Khanna

**Affiliations:** Department of Anaesthesiology & Intensive Care, AIIMS, F 35/2 Gautam Nagar, New Delhi 49, India

## Abstract

Postoperative pain management is one of the most challenging jobs in orthopedic surgical population as it comprises of patients from extremes of ages and with multiple comorbidities. Though effective, opioids may contribute to serious adverse effects particularly in old age patients. Intravenous paracetamol is widely used in the postoperative period with the hope that it may reduce opioid consumption and produce better pain relief. A brief review of human clinical trials where intravenous paracetamol was compared with placebo or no treatment in postoperative period in orthopedic surgical population has been done here. We found that four clinical trials reported that there is a significant reduction in postoperative opioid consumption. When patients received an IV injection of 2 g propacetamol, reduction of morphine consumption up to 46% has been reported. However, one study did not find any reduction of opioid requirement after spinal surgery in children and adolescent. Four clinical trials reported better pain scores when paracetamol has been used, but other three trials denied. We conclude that postoperative intravenous paracetamol is a safe and effective adjunct to opioid after orthopedic surgery, but at present there is no data to decide whether paracetamol reduces opioid related adverse effects or not.

## 1. Introduction

Postoperative pain is a major challenge in patients undergoing orthopedic surgery. Effective treatment of postoperative pain by multimodal approach is important as pain can cause neuroendocrine stress responses and other harmful effects such as autonomic reflexes with adverse effects on organ function and reflex muscle spasm [1], and in children it can cause long-lasting behavioral changes [2]. Commonly used drugs to reduce postoperative pain following orthopedic surgery include opioid, nonsteroidal anti-inflammatory drugs (NSAIDs), and paracetamol. Even though opioids are considered as the primary analgesic therapy in moderate to severe postoperative pain, these drugs do not provide optimum patient satisfaction as they are associated with dose-related adverse effects such as sedation, respiratory depression, postoperative nausea and vomiting, pruritus, and urinary retention [3, 4]. NSAIDs are associated with many adverse effects such as gastrointestinal injury, increased operative site bleeding, renal toxicity, and bronchoconstriction [5, 6]. In addition, NSAIDs have been shown to interfere with fracture healing, bone-tendon healing, spinal fusion, and bone tendon formation [7, 8]. Paracetamol with its high safety profile in recommended dosage, lack of allergic potential and absence of contraindications in peptic ulcer diseases, hemostatic disorders, or pulmonary dysfunction has gained popularity as a complementary analgesic [9–11].

The aims of the review is to assess the evidence for the effectiveness of paracetamol compared to placebo or no treatment, for postoperative pain relief, in terms of opioid consumption in patients undergoing orthopaedic surgery.

## 2. Methods

Published prospective human clinical trials which compared intravenous paracetamol with placebo or no treatment for postoperative pain management after orthopedic surgery have been included in this study.

### 2.1. Date Source and Search Method

We did an electronic search in the following database: PubMed, PubMed Central, EMBASE, and Scopus with the key words “*paracetamol,*” “*orthopedic,*” and “*orthopaedic*” to find out the eligible clinical trials on May 3rd, 2013. The search strategy in PubMed has been mentioned in Supplementary Materials available online at http://dx.doi.org/10.1155/2013/402510. References from the primary search result were again manually searched for potentially eligible trial.

### 2.2. Study Selection

Published prospective randomized human clinical trials that compared intravenous paracetamol with placebo or no treatment for postoperative pain management after orthopedic surgery have been included in this study. We did not impose any language restriction on the search strategy. Studies that have been done either in adult or pediatric population have been included in this review.

### 2.3. Exclusion Criteria

Clinical trials where paracetamol has been compared with other NSAIDs or any other drug or in surgical populations other than orthopedic surgery were not included in this review. We also excluded studies where a postoperative regional analgesia technique was used as a part of multimodal regimen. We have included studies where a single injection subarachnoid block has been used but no postoperative regional regimen was used. A single injection subarachnoid block usually provides analgesia for around 3-4 hrs, thereby unlikely influencing the postoperative pain score over a period of 24 hrs or cumulative morphine consumption.

### 2.4. Data Collection

Potentially eligible trials were manually searched to determine their eligibility in this review from the abstract. We collected the required data from the full text of the trials. Two authors independently (DKB, PK) extracted all data from the eligible trials. Initially, all data were tabulated in Microsoft Excel TM spread sheet. We did not ask the author(s) for any unpublished data.

### 2.5. Data Items

The following data were extracted from the eligible trials: name of the first author, year of publication, methods of randomization and blinding, study population, protocol of study drug administration, postoperative opioid consumption, and pain scores. All the extracted data were expressed in a Microsoft Excel spreadsheet.

Primary endpoint of our review is whether intravenous paracetamol reduces postoperative morphine consumption or not and provides better pain scores or not. Secondary endpoint was to find out effects of paracetamol on reduction of opioid-related adverse effects.

A quantitative meta-analysis was not possible as patients were undergoing different types of surgeries and dosing. Schedule of the study drug was also different.

## 3. Results

**Figure 1:**
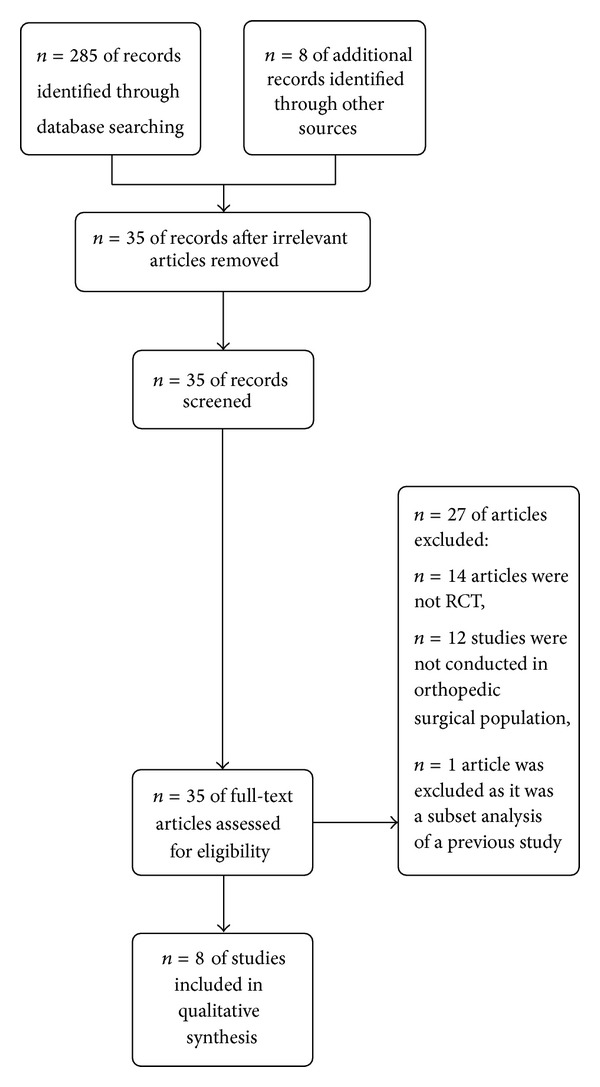
PRISMA flow diagram of study selection.

Electronic database searching resulted in 293 articles. We again manually searched all those trials in the title and abstract to find out eligible trials for this systematic review. Finally, eight prospective clinical trials were included in this analysis. We excluded a subset analysis of three clinical trials by Jahr et al. [12]. Details of search strategy have been furnished in Figure 1.

Khalili et al. [13] compared the efficacy of preemptive or preventive intravenous paracetamol with placebo in patients undergoing lower extremity orthopedic surgery under spinal anaesthesia. In this study, the control group received 100 mL of intravenous normal saline as a placebo. The preventive acetaminophen group received 100 mL normal saline and 15 mg/kg of acetaminophen prior to skin closure. The preemptive acetaminophen group received 15 mg/kg of intravenous acetaminophen combined with 100 mL of normal saline half an hour preoperatively. They recorded pain with the verbal rating scale and assessed 5 minutes before spinal anesthesia and 6, 12, 18, and 24 hours after surgery. Total rescue meperidine consumption by each patient during the first 24 hours after surgery was also recorded. Both regimens of paracetamol provided superior analgesia 6 hrs after surgery than placebo did but not in other time points. All patients in the control group, 19 (76%) in the preventive acetaminophen group, and 17 (68%) in the preemptive acetaminophen group received rescue analgesics (*P* = 0.010). They also found that average meperidine consumption during the first 24 hours postoperatively was higher in the control group than in the preemptive acetaminophen group (42 mg versus 23 mg). The adverse effects in the paracetamol treated patients were minor and infrequent, and no difference was found from the placebo in terms of adverse effects.

Hiller et al. [14] in 2012 assessed the efficacy of intravenous acetaminophen 90 mg/kg/day, adjuvant to oxycodone, after major spine surgery in children and adolescents. All the patients included in this study received oxycodone 0.1 mg/kg IV followed by an infusion of 10 *μ*/kg/h and then randomized into two groups. In the acetaminophen group, patients received 30 mg/kg IV acetaminophen infusion for 15 minutes, with a maximum dose of 1.5 g. In the placebo group the same volume of placebo was administered. Once the patients were fully awake oxycodone infusion was discontinued, and then it was administered by standard PCA pump. The VAS score was found to be significantly lower in acetaminophen group (39%) when compared to placebo group (72%) (*P* < 0.05). No significant difference was found in oxycodone consumption during the 24 h postoperative period between two groups.

Sinatra et al. [15] found that the sum of pain intensity differences over 24 hours was in favor of IV acetaminophen compared with placebo after orthopedic surgery.

Another study [16] compared the efficacy of single or repeated doses of IV acetaminophen 1 g with that of propacetamol 2 g and placebo for postoperative analgesia in patients undergoing total hip or knee replacement surgery under general or regional anesthesia. Active treatment groups had better pain relief when compared to placebo group (*P* < 0.05). Median time to first morphine rescue was also longer in active treatment groups (IV acetaminophen: 3 h; propacetamol: 2.6 h; and placebo: 0.8 h). Intravenous acetaminophen and propacetamol significantly reduced morphine consumption over the 24 h period. The total morphine doses received over 24 h were 38.3 ± 35.1 mg for intravenous acetaminophen, 40.8 ± 30.2 mg for propacetamol, and 57.4 ± 52.3 mg for placebo, corresponding to decreases of −33% (19 mg) and −29% (17 mg) for intravenous acetaminophen and propacetamol, respectively.

Hynes et al. [17] assessed the analgesic efficacy and safety of intravenous paracetamol, administered as propacetamol, in comparison with placebo and intramuscular diclofenac in patients with postoperative pain. However, we here only reviewed the comparison between paracetamol and placebo. In this randomized double blind study, 120 patients undergoing hip arthroplasty under spinal anaesthesia were included. The patients received either two administrations of propacetamol 2 g intravenously, 5 h apart (*n* = 40), one single administration of diclofenac 75 mg intramuscularly (*n* = 40), or placebo (*n* = 40). They found that total pain relief score (TOTPAR) over first five hours was significantly more in paracetamol group than in placebo (717 ± 264 for propacetamol, versus 471 ± 279 for placebo). A significantly more number of patients in placebo group requested for rescue analgesia both at 5 hr (72.5% versus 27.5%) and 10 hr (82.5% versus 47.5%) than paracetamol group. They reported twenty-three adverse events in 15/40 (37.5%) patients in the propacetamol group and 11 adverse events in 8/40 (20%) patients in the placebo group. The authors mentioned that the higher rate of adverse events in the propacetamol group was attributed to a higher incidence of injection site pain. They also found that changes in liver function tests were similar in paracetamol and placebo group.

The efficacy of IV propacetamol in combination with morphine administered by PCA was compared with IV placebo which has been assessed in patients undergoing spinal fusion surgery [18]. Patients were given either an IV injection of 2 g propacetamol or IV placebo every 6 hours for 3 days after surgery. The relief of pain was similar in both groups, except at 40 and 56 hours at which the pain scores were lower in patients receiving propacetamol (*P* < 0.01 and *P* < 0.05, resp.). The cumulative dose of morphine at 72 hrs was smaller in the propacetamol group than in the placebo group (60.3 ± 20.5 versus 112.2 ± 39.1 mg; *P* < 0.001). They also reported that most patients in the placebo group obtained a greater degree of sedation on postoperative day 3 (*P* < 0.05).

Peduto et al. [19] found that four intravenous infusions of 2 g propacetamol cause 46% reduction in PCA morphine consumption compared to placebo (9.4 ± 8.5 mg versus 17.6 ± 12 mg; *P* < 0.001). The evolution of pain intensity was similar in the two groups, but efficacy of treatment was rated significantly better by patients receiving the combination propacetamol + PCA morphine (87% of “good”/“excellent” ratings versus 65%; *P* = 0.01). Propacetamol has been evaluated in patients undergoing knee ligamentoplasty [20]. The 24 h morphine consumption was found to be significantly lower in propacetamol group (number of 1 mg boluses: 14.7 ± 11.3 versus 23.2 ± 13.8, *P* = 0.01; PCA usage: 26.4 ± 12.3 mg versus 34.6 ± 15.4 mg, *P* = 0.03; and PCA usage + titration: 34.5 ± 12.7 mg versus 43.1 ± 15.9 mg, *P* = 0.02). However, there was no difference in pain scores between the two groups.

Granry et al. [21] evaluated the effects of a single IV infusion of 30 mg kg^−1^ propacetamol (i.e., 15 mg kg^−1^ acetaminophen) with a single injection of placebo in children after limb surgery. Efficacy was assessed on pain scores rated on a four-point verbal scale, a five-point visual scale (faces), and a four-point relief verbal scale before administration (T0) and 0.25, 0.5, 1, 2, 3, 4, 5, and 6 hrs after administration. At the end, the global efficacy was rated by the physician on a five-point verbal scale. No difference existed in the first 30 minutes after infusion, but after up to 6 hrs, both visual and verbal pain scores were significantly lower in paracetamol group. The final efficacy evaluation showed 54.5% good or very good results in paracetamol group versus 33.3% in placebo group. The findings of the previous studies have been summarized in Table 1.

## 4. Discussion

The ideal way to treat postoperative pain is by a multimodal therapeutic approach [1, 22, 23]. This systematic review of randomized controlled trials provides an insight on the role played by paracetamol in postoperative pain management as a part of multimodal approach in patients undergoing orthopedic surgery.

Five clinical trials [13, 16, 18–20] reported that there is a significant reduction in opioid consumption in the postoperative period. When patients received an IV injection of 2 g propacetamol, reduction of morphine consumption up to 46% has been reported [19]. However, one study [14] did not find any reduction in opioid requirement after spinal surgery in children and adolescents. It is worth mentioning that the authors used intraoperative remifentanil infusion that may contribute to opioid induced hyperalgesia and the study population was also small, 36 only. They also calculated the sample size on basis of findings from a study done on adult patients. Moreover, it has been shown that children undergoing scoliosis surgery require significantly more postoperative opioid than others [24]. Studies, that reported a significant decrease in opioid consumption were done in adult population. Three of them were done in lower limb surgeries [13, 15, 19], one in a mixed orthopedic the surgical population [20] and, rest in spinal surgery [18]. The study, which was done in spinal surgery [18], did not show a reduced opioid consumption in first 8 hrs after surgery, but after up to 72 hrs, there was a significant reduction in opioid consumption. Again failure to reduce opioid consumption in postoperative period does not necessarily imply the failure of a drug, rather quality of pain relief in terms of patients' satisfaction and pain scores should also be taken into consideration.

Six clinical trials reported a better pain score when paracetamol has been used [13–17, 21], but other three trials [18–20] denied. The duration of action of single dose intravenous paracetamol is around 4–6 hrs, as Khalili et al. [13] found a favourable pain score only at 6 hrs. Previous systematic review found that acetaminophen combined with PCA morphine induced a significant morphine-sparing effect (mean difference 9 mg, 95% CI 3–15 mg) but did not change the incidence of morphine-related adverse effects in the postoperative period or patient's satisfaction [25], and a single dose of both IV propacetamol and IV paracetamol provides around four hours of effective analgesia for about 37% of patients with acute postoperative pain [26, 27]. Another systematic review in 2010 found that paracetamol along with PCA after major surgery reduces mean morphine consumption of 6.34 mg (95% CI 3.65–9.02) in 24 hrs. But they also did not find any difference in postoperative nausea and vomiting [28]. However, use of NSAIDS and COX-2 inhibitor causes a decrease in morphine consumption and decrease in PONV also. A previous meta-analysis by Elia et al. [29] in 2005 failed to demonstrate any benefit of intravenous paracetamol on postoperative pain score over morphine PCA either at individual study level or at pooled analysis level. But they also found a significant reduction in morphine consumption by an average of 8.3 mg in 24 hrs.

It is worth mentioning that none of the previous reviews specifically addressed orthopedic surgical population. None of the studies reported whether paracetamol reduces opioid-related adverse effects or not. Only one study reported that there was significant more sedation when paracetamol was not used on postoperative day 3. Reported adverse effects from paracetamol are mild and not associated with serious hepatic or renal consequences. One study [17] reported more adverse effects in paracetamol group; however, they attributed it to injection site pain only.

So, we conclude that postoperative intravenous paracetamol is a safe and effective component of multimodal analgesic regimen, and it reduces postoperative opioid consumption after orthopedic surgery, but at present there is insufficient data to decide whether paracetamol reduces opioid-related adverse effects or not.

## Supplementary Material


*(*“acetaminophen”*[*MeSH Terms*]* OR “acetaminophen”*[*All Fields*]* OR “paracetamol”*[*All Fields*]*) AND *(*“orthopaedic”*[*All Fields*]* OR “orthopedics”*[*MeSH Terms*]* OR “orthopedics”*[*All Fields*]* OR “orthopedic”*[*All Fields*]*).

## Figures and Tables

**Table 1:** Summary of findings from different studies.

Author	Type of surgery	Treatment groups	Duration & timing	Outcome measures	Analgesic outcome	Opioid requirement
Khalili et al., 2013 [13]	Lower extremity surgery	15 mg/kg IV paracetamol	Preventive group: Before skin closure Preemptive group: 30 min preoperative	Pain (VRS) 5 minutes before spinal anesthesia and 6, 12, 18, and 24 hours after surgery, 24 hr meperidine consumption	Lower pain score in both preemptive and preventive acetaminophen groups at 6 hours	Opioid consumption lowest in the preemptive acetaminophen group

Hiller et al., 2012 [14]	Spinal surgery in children and adolescents	30 mg/kg IV acetaminophen infusion for 15 minutes, with a maximum dose of 1.5 g	At the end of surgery and thereafter twice at 8-hour intervals	VAS Score PCA opioid requirement	VAS score significantly lower in acetaminophen group (39%) compared to placebo group (72%) (*P* < 0.05)	No significant difference was found in oxycodone consumption during the 24 h postoperative period

Hynes et al., 2006 [17]	Hip arthroplasty	Propacetamol 2 g intravenously,	Two dosages, 5 h apart	Before each drug administration, for the 5 h following each study treatment administration and for the total study duration of 10 h	Significantly better pain relief with paracetamol in comparison to placebo	Significantly more number of patients in placebo group requested for rescue analgesia both at 5 hr and 10 hr

Sinatra et al., 2005 [16]	Total hip or knee replacement surgery	Acetaminophen 1000 mg Propacetamol 2000 mg Placebo	Single and repeated doses, postoperative	Pain relief (0–5) Morphine usage (PCA)	Better pain relief when compared to placebo group	Median time to first morphine rescue was also longer, reduced morphine consumption over the 24 h period

Hernández-Palazón et al., 2001 [18]	Spinal fusion surgery	Propacetamol 2000 mg Placebo	Repeated doses, postoperative	Pain intensity (VAS) Pain intensity (VRS) Morphine usage (PCA)	The relief of pain was similar at most time points	Morphine consumption was found to be 46% lower

Delbos and Boccard, 1995 [20]	Knee ligamentoplasty	Propacetamol 2000 mg Placebo	Repeated doses, postoperative	Pain intensity (VAS) Pain intensity (VRS) Morphine usage (PCA)	No difference in pain score	At 24 h, morphine consumption was found to be significantly lower

Peduto et al., 1998 [19]	Total hip arthroplasty	Propacetamol 2000 mg Placebo	Repeated doses, postoperative	Pain intensity (VAS) Pain intensity (VRS) Morphine usage (PCA)	Pain intensity was similar	Reduction in PCA morphine consumption

Granry et al., 1997 [21]	Limb surgery in children	30 mg·kg^−1^ propacetamol	Single injection	Visual and verbal pain scale	up to 6 hrs, both visual and verbal pain scores were significantly lower in paracetamol group	
